# Smartphone apps in rare disease care: a Dutch perspective on effective implementation

**DOI:** 10.3389/fdgth.2025.1664110

**Published:** 2025-11-17

**Authors:** Ilse H. J. Willemse, Marit Mol, Rozanne J. A. van Diggelen, Pieter van den Haak, Bart P. C. van de Warrenburg

**Affiliations:** 1 Department of Neurology, Research Institute for Medical Innovation, Radboud University Medical Center, Nijmegen, Netherlands; 2 Donders Institute for Brain, Cognition and Behaviour, Nijmegen, Netherlands

**Keywords:** smartphone application, digital health, rare diseases, implementation, stakeholder management

## Abstract

Smartphone-based health applications offer promising opportunities for personalized and continuous monitoring in healthcare. However, many apps remain confined to research settings and are never implemented in clinical practice. Moreover, the development and implementation of apps for rare diseases is significantly lagging behind. This perspective outlines six key themes critical to the successful development and implementation of health apps, drawing on insights from Dutch stakeholders. These include: stakeholder collaboration, development, ownership, financing, integration into hospital-based care, and patient use. Our perspective additionally discusses specific barriers, including regulatory constraints, funding challenges, and usability limitations, alongside facilitators such as co-creation with end users, early stakeholder involvement, implementation planning, and leveraging existing care networks. Specific challenges for rare diseases, such as limited patient populations, funding constraints, and difficulties in clinical validation and regulatory compliance, are also addressed, with potential solutions proposed. This perspective offers concrete recommendations to support the transition of health apps from research to clinical practice. Sustainable implementation requires early and ongoing stakeholder engagement, flexible strategies adapted to small-scale contexts, a strong focus on end users’ needs, and an impact-driven implementation plan already established at the start of development.

## Introduction

Smartphones are a promising tool for innovating healthcare due to their widespread availability, advanced sensor capabilities, and their potential to support continuous, real-world monitoring of patients. Their integrated features—such as accelerometers, gyroscopes, GPS, cameras, and touchscreens—enable the collection of objective data on mobility, behaviour, and symptom severity at home (1). This makes smartphones particularly valuable for the remote monitoring of individuals with chronic or progressive conditions, including neurological movement disorders (2). By facilitating real-time data collection, smartphone-based tools can contribute to more personalized care (3, 4). Yet, the development of such tools for rare diseases appears to be lagging behind, with far fewer options available for individuals affected by these conditions. We illustrated this imbalance in a recent scoping review (5), which identified a total of 113 apps targeting movement disorders. Of these, 82 apps (73%) were developed specifically for individuals with Parkinson's disease, while only five apps (4%) were developed for individuals with ataxia — a rare neurological movement disorder. Another key finding was that 24 apps (21%) had been evaluated in the intended context, such as at home or in clinical practice, whereas the majority remained tested exclusively in research setting Furthermore, 28 apps (25%) were available in app stores, and none had obtained the CE-certification to comply with the European Medical Device Regulation (MDR).These numbers highlight a critical gap between app development and practical implementation, which is particularly concerning when public funding was involved.

One of the main challenges in developing an app for a rare disease is that its low prevalence impacts all phases of development and implementation, including user involvement, validation, and integration into clinical care. Furthermore, securing funding for the entire pipeline, from development to implementation, is difficult for apps targeting rare diseases. An additional challenge lies in the monitoring of specific symptoms of a rare disease, as opposed to more generic domain (e.g., electronic diaries or apps measuring vital signs). While smartphones are already capable of reliably capturing vital parameters (6), registering specific disease-related features—such as changes in gait patterns in individuals with movement disorders—remains a more complex task.

In this perspective, we will further explore these challenges, which we have structured in six important themes (stakeholder collaboration, development, ownership, financing, integration into hospital-based care and patient use), and provide concrete recommendations to support future development and implementation of apps for rare diseases. These themes are based on interviews with stakeholders in the Dutch healthcare system (Supplementary Material S1). We conducted 17 semi-structured interviews with individual or paired stakeholders from patients with rare diseases, patient associations, care networks, expert centers, department of valorisation, information management, operations, health insurers, healthcare institutes, and eHealth professionals. Interviews were recorded, transcribed, and thematically analyzed. The interviewer underwent training in qualitative research methods, including sessions on reflexivity and managing personal biases, and an observer was present to review and discuss the interviews afterward to further reduce potential bias. Supplementary Material S2 presents an overview of the most frequently reported barriers and facilitators for each theme, indicating the stakeholder group that raised them most prominently and including illustrative quotes. Though many insights are relevant beyond the national context and applicable to international digital health efforts.

## Stakeholder collaboration

**Figure 1:**
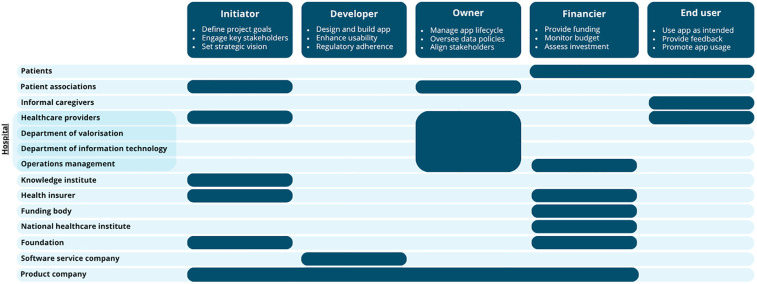
An overview of the most frequently mentioned stakeholders and their specific role(s) during the development and implementation of an app. For each role, three example responsibilities are provided.

Identifying all relevant stakeholders is a critical and early step in the development and implementation of healthcare apps. Stakeholder identification methods include reviewing literature, expert recommendations, and snowball sampling, where current stakeholders suggest additional relevant participants (7). Figure 1 presents an example of an overview of key stakeholders involved in the development and implementation of an eHealth monitoring app for somatic care, along with their potential roles. The roles presented are indicative and should be adapted in collaboration with stakeholders to fit the app's intended context.

Once key stakeholders and their roles are identified, effective collaboration is essential for successful implementation. Partnerships between hospitals or knowledge institutes and companies can strengthen this process. Hospitals contribute clinical expertise, patient engagement, and research capacity (university medical centers), while companies bring knowledge of regulations, commercial strategies, and sustainable business models.

Ongoing stakeholder involvement is crucial to ensure the app meets user needs, complies with regulations, and remains financially sustainable. However, managing stakeholders takes time and is particularly challenging in rare diseases, where insurers and investors may lack familiarity with the condition, and patient populations and organisations are smaller and less professionally supported. Care networks can support knowledge sharing and outreach; even in the absence of disease-specific networks, related networks may be used, as professionals often work across overlapping conditions (8).

## Development

The first crucial step in developing a healthcare application is creating an impact-driven strategic plan centred around the core question and specific needs of the end users, whether these are healthcare providers and/or patients. This plan should emerge from collaborations between researchers and stakeholders through methods such as co-design and co-creation, ensuring that the outcome is both scientifically robust and socially relevant (9). An impact-driven plan starts with identifying the individuals or organizations who will use the app. Interactions with rare disease patients is essential to tailor the development process so that the app aligns with their needs. A Business Model Canvas (BMC) (Supplementary Material S3) helps mapping the core components of the initiative, enabling targeted management of societal value and feasibility (7). This canvas can be continuously in transition depending on the development or implementation phase of the app.

In addition to practical feasibility and economic viability, scientific validity remains essential throughout app development. Developing apps for rare diseases faces extra challenges due to limited data, low commercial interest, and scarce public awareness, which complicate funding. Using existing apps or platforms saves time, reduces costs, and eases regulatory compliance as they are often clinically validated and supported by established infrastructure. Small patient populations require tailored measurement tools and limit large-scale testing, so alternatives include testing with patients having similar symptoms or healthy controls to evaluate usability. Incorporating generic but relevant features can increase flexibility and support broader acceptance and successful implementation.

Beyond the specific challenges of rare diseases, there are universal aspects—such as data management—that are critical for all health apps. Clearly defining what data are collected and how they are handled helps minimize redundancy and ensures compliance with legal standards. Creating a data management plan, already early in the development process, will enhance efficient and correct data processing in next stages.

## Ownership

Clear agreements on ownership of an app, including responsibilities related to technical maintenance, legal compliance, clinical content, and patient management, are essential for sustainable implementation. Ownership can generally take two forms, each with distinct implications for long-term sustainability and integration.

One common option is business ownership, where a product company such as a start-up from a hospital or research institute, or an established digital health platform provider, brings the app to the market and supports its integration into healthcare. Many assume that launching an app is primarily a technical challenge. However, it is a misconception that a software service company alone can bring an app to market; this role requires a dedicated product company capable of maintaining a sustainable business model over time. A product company takes ownership of the app, manages its lifecycle and builds a business model around it. In contrast, a software service company typically builds digital solutions on a project basis without long-term responsibility for commercial success or market adoption. However, success depends on market uptake, which can be limited in rare diseases, making this approach financially risky. We will elaborate on this in the theme “Financing”.

Alternatively, a hospital or knowledge institute may retain ownership of the app, especially if development started within the institution. This can support integration into routine care and improve access when embedded in standard pathways. Hospital ownership enhances collaboration between developers, researchers, and clinical staff, improving further development and evaluation. Additionally, cost management remains within the healthcare provider's control, supporting funding negotiations with insurers or grant agencies. However, budgetary constraints in hospitals can limit financial capacity to maintain or expand implementation. Also, experience and expertise for ownership, and the focus and drive to work toward broader implementation and optimization in support of a viable business case are often lacking in hospitals and knowledge institutes. Lastly, care for rare diseases is often centralized in expert centres that may hold full ownership. Patients in general hospitals may lack access to an app if those do not prioritize or allocate resources for rare disease care. Early stakeholder engagement is essential to ensure equitable access for all patients.

## Financing

A sustainable business model is essential to finance the implementation and maintenance of health apps. The most appropriate finance method varies per app and depends on factors such as the app's functionality, target users, and intended purpose.

First, reimbursement by health insurers can cover app expenses, enabling patients and healthcare providers to use the app without direct payment. This is feasible when the app fits within reimbursable healthcare packages, such as the Dutch telemonitoring performance, which serves as an example of a national reimbursement scheme for remote patient monitoring. Defined by the Dutch National Health Care Institute (Zorginstituut Nederland), this performance allows healthcare providers to receive reimbursement for remote monitoring services that meet criteria for safety, effectiveness, and integration in care pathways. Insurers require evidence of clinical effectiveness, cost-effectiveness, and feasibility before approving reimbursement. These evidence requirements are usually not fixed numerical cut-offs (e.g., a specific effect size), but rather relate to the quality, type, and relevance of the supporting evidence. Generally, apps that improve care quality but increase costs may face reimbursement challenges, unless they demonstrably reduce overall healthcare burden (e.g., by lowering staffing needs or workload). For rare diseases, reimbursement is particularly challenging due to limited evidence and small patient populations. However, research tailored to these populations and leveraging existing data or international evidence can support insurer acceptance.

Secondly, apps may be financed directly by end users through one-time purchases or subscription fees. User willingness to pay depends on user's experienced added value, such as improved quality of life or symptom management. Ethical considerations require that patients should never be forced to pay for essential treatments via apps, as this risks creating inequities in access, especially affecting socioeconomically disadvantaged groups. This method of financing an app is especially difficult for rare diseases due to small markets and limited financial capacity of patient groups, potentially limiting sustainable development and broad access.

**Figure 2:**
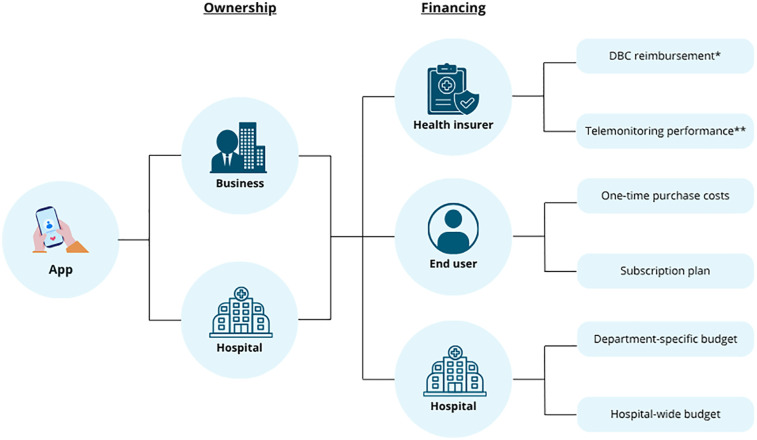
An overview of the various potential pathways for ownership and financing of a healthcare app within the Dutch healthcare system. *DBC (Diagnosis Treatment Combination) reimbursement refers to the Dutch system of bundled payments for diagnosis and treatment, where healthcare providers are reimbursed on a predefined care pathway for a given diagnosis. **In The Netherlands, the telemonitoring performance is a reimbursable healthcare service that allows providers to claim costs specifically for the remote monitoring of patients as part of chronic care management. Created using Canva.

Finally, healthcare providers may fund app implementation through departmental budgets if clear benefits, such as reduced workload, shorter consultations, enhanced disease insight, or personalized care, are experienced or proven. Benefits to patients may include fewer hospital visits and better daily support. However, hospitals often face budget constraints, making it difficult to allocate funds without proven cost-effectiveness or reallocation of existing resources. Additionally, financial benefits may not affect the investing department, complicating cost-benefit alignment. Integrating apps into existing certified digital health platforms can reduce implementation costs and reduce the risks related to small target populations by using shared infrastructure. Figure 2 presents the potential pathways for ownership and financing of an app in the Netherlands. Comparable pathways to the Dutch DBC (Diagnosis Treatment Combination) reimbursement exist in other countries, such as bundled payment models in the United States or tariff-based care pathways in other European systems, though accessibility and eligibility criteria may vary depending on national policies (10, 11).

## Integration into hospital-based care

Successful integration of an app into hospital workflows is most feasible when it aligns with or replaces parts of existing care pathways within hybrid care pathways. This benefits both patients and healthcare providers by improving usability, communication, and collaboration. However, integration is more difficult for rare diseases, as they are often not included in treatment guidelines and standardized protocols.

For successful integration, it is essential to scientifically validate the app's effectiveness. The app must be safe, reliable, and accurate for clinical use. Key effectiveness indicators include clinical accuracy and the potential to reduce the workload for healthcare professionals. Any digital solution should alleviate the pressure on a healthcare system. This could be facilitated by providing targeted training for healthcare professionals. Education about the added value increases motivation and supports behaviour change, which is a challenge in implementation of healthcare innovations.

An important barrier to consider is regulatory compliance. Health apps must meet strict requirements concerning safety, privacy, and data protection. Integrating the app into existing hospital IT infrastructure is complex. Furthermore, data sharing between hospitals remains difficult due to legal frameworks, hindering multi-site implementation of the app. Healthcare professionals also face practical challenges. While apps can collect large volumes of data, clinicians must have the time and capacity to process and interpret this information.

Moreover, the rapid pace of technological advancement complicates integration. Both hospital IT systems and apps themselves are continuously evolving, which may result in misalignment by the time research and development are complete. Proactive collaboration with relevant stakeholders who possess the technical and clinical expertise to anticipate such developments is needed for ensuring successful and sustainable integration.

## Patient use

Successful app development and implementation must align with user needs. Patients are more likely to engage when an app feels relevant and valuable to their personal situation. Clear communication about the app's purpose, content, and data use is essential, especially when active participation is needed. Patient organizations can support implementation by sharing reliable information and promoting the app. Users often require personal support, which can be provided by healthcare professionals, helpdesks, or trained staff within care networks. Even for rare diseases, existing networks for related conditions can support training, implementation, and knowledge exchange.

The usability of an app is a determining factor influencing adoption and long-term use, particularly among patients. However, its importance is often underestimated during development. Key requirements include compatibility with both iOS and Android devices, availability in the user's native language, and the use of clear, jargon-free language, ideally at a B1 reading level. Failing to meet these basic standards can result in user frustration and disengagement. A simple login process, low battery consumption, and a visually clear interface with minimal required actions are highly valued examples. Automated reminders or notifications are also seen as supportive and motivating. These highlight the need for a user-centred design approach.

Maintaining motivation for long-term or repeated use of apps is another key challenge. Patients are more likely to remain engaged when they perceive direct personal benefit. Long-term engagement can be enhanced through positive feedback, visualized progress, and motivational features such as gamification. However, for some users, app use can be burdensome, as it is a constant reminder of their illness. Additionally, limited digital literacy, particularly among older adults, presents a significant barrier in long-term engagement. In such cases, personalized support and accessible help services are essential to enabling continued use.

Finally, ethical principles such as safety, autonomy, and equity must be considered during the implementation of health apps. Use or testing of the app should not burden or endanger patients. Apps must be accessible to all, regardless of socioeconomic status or disease rarity, in order to prevent health disparities. Personal data must be handled securely and accessed only by authorized individuals. Transparent and honest communication about the app's purpose and data use is essential, along with asking informed consent. The app design and communication must be respectful, inclusive, and non-discriminatory, while ensuring that users retain the freedom to choose whether or not to use the app.

## Discussion and conclusion

The development and implementation of health apps, particularly for rare diseases, faces unique challenges, but it also presents opportunities to promote more equitable, personalized, and sustainable care. This perspective has highlighted the possible barriers and facilitators to support this process through six themes.

Successful app development and implementation relies on early identification and ongoing collaboration of key stakeholders. Active involvement of end users must begin at the earliest stages of development. This co-creation approach increases the likelihood that the final app will meet real-world needs and promote long-term engagement. Furthermore, these insights emphasize that an impact-driven plan is essential to ensure scientific validity, practical feasibility, and economic viability. Creating a BMC early on can support this process by outlining the core components.

Clear ownership arrangements are vital for the sustainable implementation of health apps, with options including business or hospital ownership. While business ownership can provide resources for a sustainable business model and growth, hospital ownership may facilitate care integration and accessibility, especially for rare diseases, but requires early stakeholder engagement to ensure equitable access across all hospitals. Sustainable financing of health apps can be facilitated via insurer reimbursement, payment by patients, or hospital funding. It is important to highlight that inequities in access to care should always be avoided, especially if end users are required to pay for a health app. These findings also emphasize that there is no one size fits all approach for app implementation. The most appropriate route must be based on the app's purpose, the patient and disease context, and available resources. Decisions regarding ownership and financing should be made collaboratively, considering both the needs of end users and the sustainability of the initiative. The integration of health apps into hospital settings remains complex due to strict regulatory demands, challenges with data interoperability, time constrains of healthcare professionals, and the rapid pace of technological advancements. These barriers require multidisciplinary collaboration to ensure an app can be safely, effectively, and sustainably integrated within already existing care pathways. Reusing or adapting existing apps and platforms could pose an effective solution. This strategy could reduce regulatory barriers, lower development costs, and accelerate the implementation process, advantages that are particularly relevant for rare diseases where limited resources and lower patient numbers make successful implementation less feasible.

Another critical factor for successful implementation of an app is patient engagement, which depends on clear relevance, usability, and ongoing personal support, with patient organizations and care networks playing key roles in promotion and training. Additionally, ethical considerations, including transparency, data security, equity, and respect for autonomy, are essential to ensure safe, inclusive, and sustained app use.

To conclude, while there is no universal preferred route for development and implementation of a health app, prioritizing end users’ specific needs by early stakeholder involvement increases the chances of meaningful and successful implementation in clinical practice. Furthermore, active stakeholder involvement throughout the entire process and consideration of all clinical, patient, financial, legal, organizational, and technical aspects using a BMC can ensure that apps do not remain confined to research settings but are integrated sustainably in healthcare. For rare diseases, it is essential to tailor implementation strategies to smaller-scale contexts, engage care networks, and prioritize accessible and user-centred development and implementation. These recommendations should be considered in light of the study's context, including the relatively small and interconnected stakeholder groups, the focus on physical monitoring apps, and the absence of formal effectiveness or economic evaluation.

To embed these principles into practice, the main recommendation is for researchers to establish an impact-driven implementation plan at the very start of developing a new digital application. Rather than allocating research budgets to the creation of custom built applications developed for temporary use within a single study, often by small software service teams with limited continuity, researchers should first examine whether existing validated platforms can be adapted to serve the research objectives. This approach promotes sustainable innovation and increases the likelihood of integration into routine care.

## Data Availability

The datasets presented in this article are not readily available due to privacy or ethical restrictions. Requests to access the datasets should be directed to the corresponding author.
