# Supplementary material for: Smartphone apps in rare disease care: a Dutch perspective on effective implementation
Source: Front Digit Health. 2025 Nov 17;7:1664110. doi: 10.3389/fdgth.2025.1664110 (PMC12666554; doi:10.3389/fdgth.2025.1664110)
Supplement: Supplementary file 1 [file Datasheet1.pdf]

Supplementary materials

Supplementary material 1: Methods

A qualitative approach was used to collect relevant input for this perspective. Relevant stakeholders were identified through expert recommendations and participating stakeholders. The participants included three patients with rare diseases, two patient associations, two experts from a care network, one expert centre, two valorisation experts, one information management expert, one operations management expert, one health insurer, one healthcare institute, two eHealth experts, and five experienced professionals from three different companies that have developed and implemented applications within Dutch healthcare.

An interview guide with open-ended questions was developed to conduct semi-structured interviews, covering topics such as development, implementation, ownership, acceptance, ethical considerations, and relevant stakeholders. The questions were based on an evaluation of the PROSPAX project outcomes in collaboration with the patient association AtaxiaUK. Insights from this evaluation informed the development of the interview guide, which was adapted as needed—for example, patient interviews included questions more specifically tailored to the patient perspective.

All stakeholders were interviewed individually or in small groups (up to two people). Group interviews were conducted when multiple experts belonged to the same stakeholder group. In total, seventeen interviews were held. The interviews were thematically analysed to identify overarching themes, main barriers and facilitators, and recommendations for development and implementation.

Supplementary material 2:

Key stakeholder groups	Frequently mentioned facilitators / barriers	Exemplar quotes
**Theme 1: Stakeholder collaboration**		
eHealth professionals<br>Care network<br>Health insurers<br>Expert centers	Identification relevant stakeholders<br>Involvement of end users<br>Collaboration with company that markets app<br><br>Stakeholders less familiar with disorder<br>Lack of healthcare network for implementation<br>Smaller patient associations.	*"It is important to involve end users as this helps to identify and prevent problems that would not have been anticipated without their active participation."*<br><br>*"For rarer conditions, the first challenge is often to determine whether a patient organization even exists and how to reach the patients themselves."*
**Theme 2: Development**		
Patient association<br>eHealth professionals<br>Information management	Testing app with different target group<br>Identifying generic elements in app<br>Development of a Business Model Canvas<br><br>Smaller target population<br>Funding for research<br>Specific measurement elements	*"We observed that many older adults don't realize they can scroll for more features, which can be tested with the comparable target group rather than patients specifically."*<br><br>*"With such a small sample size, one outlier can really skew the results. So you have to make sure there are enough people in each group to get a reasonably representative picture."*
**Theme 3: Ownership**		
Information management<br>Department of valorisation<br>eHealth professionals	Defining responsibility for data<br>Drafting contracts<br><br>Legislations and regulations	*"You have to make very clear agreements: who are the owners? Who has access to the data?"*<br><br>*"You have to comply with all legal requirements, with all the associated risks. And the risks that apply here are that the data might not be collected properly, leading to incorrect outcomes."*
**Theme 4: Financing**		
Health insurers<br>eHealth professionals<br>Department of valorisation<br>Expert centers<br>Operations management	Smaller server capacity means lower costs<br>Developing a business plan<br>DBC reimbursement and telemonitoring performance*<br><br>Appropriate research into cost-effectiveness<br>Small market<br>Sustainable business model	*"The more niche the population, the weaker the business case tends to be."*<br><br>*"How can you create a business case to make it financially viable? Could it be funded from existing care if it reduces the workload for healthcare professionals? Or does it provide new insights that help improve treatment? Based on those improvements or shifts in care, you can then decide what resources the department is willing to invest."*
**Theme 5: Hospital integration**		
eHealth professionals<br>Information management<br>Expert centers<br>Operations management	Implementation in existing care pathway<br>Scientific evidence as an incentive for healthcare professionals<br>Reduction of workload<br><br>Rare disease not included in treatment guidelines<br>Interpretation of collected data<br>Rapidly evolving technology	*"When data are collected through digital monitoring systems, it is essential that clinicians can interpret them correctly — something that is often lacking in practice."*<br><br>*"Many healthcare professionals are already overwhelmed with extra tasks, logging into yet another web application to enter data is not ideal. Such systems should integrate seamlessly with the electronic patient record, but that's often a real challenge."*
**Theme 6: Patient use**		
Patients<br>Patients associations<br>eHealth professionals<br>Care networks<br>Expert centers	Added value for the end user<br>Clarity about what is measured and why<br>Promotion by patient associations<br><br>Long-term motivation<br>Confrontation with illness<br>Lower digital skills	*"Downloading an app is easy; staying motivated to use it regularly is the real challenge."*<br><br>*"I can tell that my disease is getting worse. That's also why I prefer not to see the neurologist too often, they'll only notice my decline, and I'd rather not be reminded of that."*

* DBC (Diagnosis Treatment Combination) reimbursement refers to the Dutch system of bundled payments for diagnosis and treatment and the telemonitoring performance is a reimbursable healthcare service that allows providers to claim costs specifically for the remote monitoring of patients in the Netherlands.

Supplementary material 3: Example of a Business Model Canvas

As an example, the BMC for a telemonitoring app has here been elaborated, for the scenario in which a business owns the app and the costs are reimbursed by a health insurer. This example was chosen because, based on the interviews, this appears to be currently the most feasible implementation route for telemonitoring apps in the Netherlands.

Key Partners

- Patients
- Patient associations
- Healthcare providers
- Health insurers
- App owner (business)
- Software developers

Key Activities

- Development and maintenance of the app
- Quality assurance and validation
- Compliance with legislations and regulations
- Collaboration with health insurers
- Training and support for patients and/or healthcare providers
- User support (e.g., helpdesk)
- Monitoring of outcomes and usage
- Marketing and education
- Stakeholder management

Key Resources

- Technological infrastructure
- Intellectual property
- IT support
- Customer service/helpdesk
- Patient needs
- Medical guidelines
- Time/FTEs

Value Propositions

- Accessible health monitoring
- Personalised health advice
- Prevention of health problems
- Cost savings through prevention
- Higher quality of patient care
- Improved quality of life
- More efficient care processes
- Time savings
- Data for evaluation and research

Customer Relationships

- Accessible app
- App usability
- Personal guidance
- Automation (e.g., push notifications and automated reports or updates)
- Reward system

Channels

- Patient associations
- Healthcare institutions
- (Healthcare) networks
- Centres of expertise
- Health insurers
- Websites
- Social media
- Conferences

Customers

- Patients
- Healthcare providers
- Health insurers
- Hospitals

Costs

- Development costs
- Maintenance and management costs
- Marketing costs
- Certifications
- Health insurer costs (e.g., administrative costs and cooperation agreements)
- Personnel costs (time/FTEs)

Revenues

- Reimbursements from health insurers
- Investments
- Grants
